# Computer Vision in Analyzing the Propagation of a Gas–Gunpowder Jet †

**DOI:** 10.3390/s22010006

**Published:** 2021-12-21

**Authors:** Irina G. Palchikova, Igor V. Latyshov, Evgenii S. Smirnov, Vasilii A. Vasiliev, Alexander V. Kondakov, Irina A. Budaeva

**Affiliations:** 1 Technological Design Institute of Scientific Instrument Engineering of the Siberian Branch of the RAS, 630058 Novosibirsk, Russia; evgenii.s.smirnov@yandex.ru (E.S.S.); i.budaeva@g.nsu.ru (I.A.B.); 2 Saint Petersburg University of the Ministry of the Interior of Russia, 198206 St. Petersburg, Russia; latyshov@gmail.com; 3 Volgograd Academy of the Ministry of Interior of Russia, 400089 Volgograd, Russia; v-vasiliev@inbox.ru; 4 Federal State Institution of Higher Education, Saint Petersburg Academy of the Investigative Committee of the Russian Federation, 190068 St. Petersburg, Russia; akondakov77@mail.ru

**Keywords:** gunshot residues, target, digital image processing, optical density, gas–gunpowder jet

## Abstract

A method of mathematically processing the digital images of targets is developed. The theoretical and mathematical justification and the experimental validation of the possibility of estimating the amount of gunshot residue (GSR) and determining the GSR distribution over the target on the basis of its digital image is provided. The analysis of the optical density in selected concentric rings in the images reveals the radial dependence of soot distribution in the cross section of a gas–gunpowder jet. The analysis of the optical density in selected sectors of the circle reveals the angular dependence of the soot distribution in the gas–gunpowder jet cross section. It is shown that the integral optical density averaged over a selected area in the target image characterizes the mass of GSP deposited on it. It is possible to quantify the differences in the radial and angular distributions of the thickness of the GSR layer on various targets obtained both with the help of weapons of different types at the same distances and with the help of weapons of the same type at different distances, by calculating the distribution of optical density on their digital images.

## 1. Introduction

The forensic investigation of the circumstances of an incident makes it possible to reconstruct the overall picture of the incident. The tasks of detecting residues of a close shot on objects, as well as establishing the distance of the shot by expert examination, continue to be relevant. However, forensic practice today does not have a sufficient set of mobile technical and forensic tools and methods to identify gunshot residues (GSR) on objects, and expert examination of GSR in most cases is carried out with complex and expensive equipment through the use of destructive methods of investigation. The existing methodological approach also does not fully meet the needs of practice. It is necessary to integrate innovative means and methods of expert examination into the process of analysis and assessment of GSR, including those obtained by theoretical and mathematical substantiation.

In the structure of the significant issues of expert examination of GSR, the problems of studying the parameters of the gas–gunpowder jet are of particular interest [1]. The most important parameters are the minimum diameter and the density distribution in the plane of the so-called caustics [2]. In order to detect and measure the particle density distribution and caustics of the powder jet, the jet is illuminated with a laser line perpendicular to the powder gas flow and observed by the coaxially arranged camera through the powder nozzle [2]. The system changes the relative position of the laser and the machining head several times for further measurements. Finally, the evaluation of 2000 to 3000 images shows the statistical distribution of the particles in one plane. This method allows you to find the parameters of the jet directly at the time of its outflow.

The forensic investigation of incidents involving firearms routinely involves attempts to detect gunshot GSR on samples taken from different surfaces. The results of GSR analyses are often used in crime reconstruction to determine a variety of details, including the shooting distance/trajectory [3,4,5] and the characteristics of the firearm/ammunition combination [6,7]. GSR is produced when a gun is fired and is composed of compounds from the bullet, cartridge, and firearm ([8] p. 241). These materials cool and condense on surfaces in the vicinity. A comprehensive review of analytical methods for the detection of GSR is made in [9,10]. Studies based on special methods (chromatography, atomic adsorption spectroscopy [11], mass spectroscopy [12], inverse voltammetry [13], X-ray fluorescent analysis, and several others [14] are quite difficult in hardware design, require special knowledge in the evaluation and interpretation of results, and are often destructive.

The most established and refined technique is scanning electron microscopy with energy-dispersive X-ray spectroscopy (SEM/EDX). This method determines the size and shape of the GSR particles and the elemental composition of the GSR (GSR examined for the presence of certain elements) [15]. However, this methodological approach also involves removing GSR from the target.

Among the methods of studying GSR in forensic ballistics, the diffusion-contact method, as well as the optical method of studying GSR in the visible and infrared range of the spectrum, are the most widespread [16]. Research with the diffusion-contact method [17] is based on the qualitative determination of metallization products formed as a result of high temperatures and pressures and deposited on the target [18]. This method allows you to determine most of the known metals: copper, nickel, iron, lead, and several others. However, due to the process of chemical diffusion resulting from the interaction between the target material, solvent, and substrate (fixed photographic paper), an error is introduced into the representation of the GSR distribution. This method is also destructive, which makes it impossible to re-examine the object.

Important for the forensic investigation of incidents of particular importance is the undamaged state of samples with GSR. Undoubtedly, the task of substantiation and development of nondestructive methods of examination of GSR, which can include methods of computer vision and digital photography, is relevant. To visualize the distribution of gunshot products on targets when studying GSR in the visible and infrared spectral bands, the technical means designed to solve problems of a forensic examination of documents are sometimes used [19]. However, this solution has its disadvantages. In particular, the gunshot products (and in some cases the targets) contain components with luminescence, or particles of substances that are not transparent in this spectral region, which ultimately distorts the results obtained.

Processing the digital images [20] of various objects aimed at finding their morphometric characteristics is widely used in various fields [21], e.g., in studying geological formations, in determining the shapes, structures, and sizes of the individual cells of biological tissues and organs in medical diagnostics, etc.

The use and development of computer vision methods in the tasks of expert examinations of GSR is also relevant [22]. The tasks include identifying and fixing GSR on objects, analyzing the characteristics of the main and additional gunshot traces, including measuring GSR, calculating morphology parameters [23], and the topography of their deposition. The results obtained are used in determining the direction and distance of the shot. Modern computer technology makes it possible to determine quantitative parameters of the morphology of GSR from their photographic image. However, the problem of determining the amount of GSR on a target is usually solved only by destroying the target [24].

At the same time, in optical spectroscopy, there are widely used methods for quantitative analysis of a dispersion medium, in using which the samples are not destroyed. The nondestructive method of molecular absorption spectroscopy, which serves to determine the quantitative content of the component [25,26,27] is based on the well-known Bouguer–Lambert–Beer law, which describes the proportion to which a sample transmits light of a particular wavelength. In [28], it was experimentally shown that modern digital cameras with a wide dynamic range allow construction of cytophotometric devices for determination of DNA content in the cell nucleus of Feulgen-stained samples.

The accuracy of these devices is not inferior to the accuracy of Vickers M8 scanning densitometers. Informative morphometric parameters of a cell nuclei chromatin include an integral optical density, the fraction of compact and diffuse chromatin in cell nuclei, and the area occupied by them. These parameters are found by processing the digital images of samples. The processing algorithm includes image normalization, segmentation (a determination of cell nuclei borders), and a calculation of optical density both in each pixel of the image and its integral value. In other words, modern technologies and element base allow developing computer vision methods in application to cytometry tasks according to European 1999 Prototype Reference Standard Slide (PRESS) project.

Under certain conditions, many processes of light scattering [29] are governed by relations and laws similar to the Bouguer–Lambert–Beer law. Light scattering by diluted suspensions [30] and molecules [31], as well as scattering of electron beams [32] in gas media, occur with exponential attenuation with an increase in the layer thickness and concentration of scattering agents.

In the present work, for the first time, the possibility of estimating the amount of GSR and its distribution on a target by means of its digital photo is substantiated theoretically and confirmed by experiment, that is, by nondestructive method.

The rest of the paper is structured as follows. In Section 2, concepts of the theoretical model and its mathematical description are depicted. In Section 3, calculation procedure and the specialized image processing program ImgOpinion are described. In Section 4, we explore in more detail the requirements of the experimental setup, the image processing results, and their features. In Section 5, we discuss approximations adopted in constructing the model and the limitations of the proposed method, and draw conclusions.

## 2. Mathematical Description

For a mathematical description of the problem, we proceed from the following model of the phenomenon under the constraints defined below. A list of symbols in the formulas and their explanations, which would disrupt the flow of the main text but nonetheless remain crucial to understanding formulas, can be found in Glossary.

After the shot, all components of the gas–gunpowder jet move in the form of a three-dimensional cloud. The substance of the cloud is consistently deposited on a barrier (target) aligned almost normal to the barrel of the gun. A GSR signature pattern is formed in the primary deposit area [33].

Let the following conditions be satisfied:

(i) The gas–gunpowder jet has a limited volume, i.e., a limited amount of a substance exhausts in the jet form during the shot. The total amount of substance that was not scattered and did not leave the jet before its impact onto the target is deposited on the target. The thickness *h* of the GSR layer on the target surface depends on the position (coordinate) of the target, and the substance is homogeneous. Then, the substance mass *m* on the surface area σ is directly proportional to the product σ*h*.
(ii) When the target is illuminated by visible light, light reflection, transmission, and absorption occur. The following relation is valid: *R* + *A* + *T* = *const* (*R* is the intensity reflection coefficient, *A* is the intensity absorption coefficient, and *T* is the intensity transmission coefficient). Without loss of generality, it may be assumed that *const* = 1. It should be noted that *R* and *T* also depend on the target material.
(iii) The target is made of a homogeneous, single-colored material with the reflection and transmission coefficients in terms of intensity over the target surface being constant if there is no GSR on the surface.
(iv) The local absorption coefficient *A* is directly proportional to the GSR layer thickness and, hence, to the amount of substance on the elementary area.
(v) The total area of regions where the GSR layer thickness is greater than that at which the entire incident radiation is absorbed is rather small.

If the last three conditions are satisfied, then an increase in the GSR layer thickness *h* leads to proportional reduction of the coefficients *R* and *T*; therefore, the values of these coefficients depend on the amount of substance deposited on the area.

It is the distribution of *R* or *T* that is registered by digital photo-image, depending on the method used to take the images (image in reflected or transmitted light) in the case of diffuse reflection. If the camera is tuned correctly, the total dynamic range is used, and the images are taken on the linear section of the transfer function of the photomatrix. In the case of image in transmitted light, the local brightness $I_{i}$ in each pixel of the digital image is directly proportional to $T_{i}$ and depends on the GSL layer thickness $h_{i}$ on the corresponding area of the target surface. The brightness is measured in grayscale level, and the maximum value depends on camera digitalization (256 levels if the digitalization value is 8 bits per channel). In order to examine the distribution of GSR on the target surface, nonoverlapping areas of interest can be selected in the image. Hereinafter, the subscript *i* marks the values corresponding to one pixel of the image; *i* is the number of the pixel, and in the selection area *i* varies from 1 to *N*.

The change in brightness as a function of the GSR layer thickness can be written as

(1) $$dI_{i}=-I_{i}\beta dh_{i},$$

where $dI_{i}$ is the change in the initial brightness $I_{i}$, $dh_{i}$ is the layer thickness, and β is a certain proportionality coefficient, which characterizes the properties of the target material, gunpowder, and its adhesion to the target and which is expected to depend on the light wavelength. This differential equation has a trivial solution:

(2) $$I_{i}=I_{0}e^{-\beta h_{i}},$$

where $I_{0}$ is the brightness (mean value) in the image in the regions where GSR is absent. The coefficient β is independent of $I_{i}$ and GSR layer thickness $h_{i}$. It can be determined as the thickness of the layer that ensures brightness attenuation in the image by a factor of e after light reflection from this layer.

Then, the optical density in each pixel of the image is

(3) $$D_{i}=-\ln\frac{I_{i}}{I_{0}}=\beta h_{i}.$$

Thus, the optical density in the digital image is directly proportional to the GSR layer thickness $h_{i}$ on the corresponding area of the target (or in each pixel of the selected area).

The integrated brightness over the selected area is found by summation of the left-hand and right-hand sides of Equation (2) over the selected area $\tilde{S}$:

(4) $$\sum_{\tilde{S}}I_{i}=I_{0}\sum_{i=1}^{N}e^{-\beta h_{i}}=I_{0}e^{-\beta h_{\max}}\left\{1+e^{-\beta \frac{h_{1}}{h_{\max}}}+\left.\cdots +e^{-\beta \frac{h_{i}}{h_{\max}}}+\cdots +e^{-\beta \frac{h_{N}}{h_{\max}}}\right\}\right..$$

The value of $h_{i}$ varies over the target surface. In Equation (4), $h_{\max}$ is the maximum thickness, and each of the terms is smaller than unity: $e^{-\beta \frac{h_{N}}{h_{\max}}}<1$. Then, the maximum value for the sum of the series can be estimated and it will be less than the number of pixels in the selection area—*N*:

(5) $$1+e^{-\beta \frac{h_{1}}{h_{\max}}}+e^{-\beta \frac{h_{2}}{h_{\max}}}+\cdots +e^{-\beta \frac{h_{i}}{h_{\max}}}+\cdots +e^{-\beta \frac{h_{N}}{h_{\max}}}\leq N.$$

Under these conditions, there always exists an effective thickness $h_{eff}$ at which the following equality is satisfied:

(6) $$I_{0}e^{-\beta h_{eff}}N=I_{0}e^{-\beta h_{\max}}\left\{1+e^{-\beta \frac{h_{1}}{h_{\max}}}+e^{-\beta \frac{h_{2}}{h_{\max}}}+\right.\left.\cdots +e^{-\beta \frac{h_{i}}{h_{\max}}}\right\}.$$

Combining Equations (4)–(6), we find

(7) $$\sum_{i=1}^{N}I_{i}=I_{0}e^{-\beta h_{eff}}N.$$

As a result, taking the logarithm of Equation (7), we obtain

(8) $$\ln\frac{I_{0}N}{\sum_{i=1}^{N}I_{i}}=\beta h_{eff}.$$

The product $\sigma_{i}Nh_{eff}$ is directly proportional to the mass *m* of the GSR on the selected area, where $\sigma_{i}$ is the area of one pixel:

(9) $$m=\sigma_{i}Nh_{eff}=N\cdot \ln\frac{I_{0}N}{\sum_{i=1}^{N}I_{i}}\cdot \frac{\sigma_{i}}{\beta }.$$

Thus, the integrated optical density averaged over the selected area in the image characterizes the soot mass on the selected area:

(10) $$m=ND_{\Sigma }\frac{\sigma_{i}}{\beta }.$$

The proposed approach and the present consideration allow one to detect and experimentally determine the substance distribution in the cross section of the gas–gunpowder jet by means of calculations based on the digital image of the pattern at a target.

## 3. Analyzing Procedure and Software

Methods for calculating optical density from a selected area of a digital image are well known and widely used, e.g., in biological experiments [34], and can be performed with various software and programming environments: Microsoft Office Excel, PTC Mathcad, Matlab, ImageJ [35], etc.

The calculations based on the digital image for determining the GSR amount in accordance with Equation (10) are performed in the following procedure:

(i) The value of $I_{0}$ is calculated as the mean value of brightness on the selected image area not covered by GSR.
(ii) Areas of interest are selected. To select areas of interest, the ImgOpinion interface provides for setting the coordinates of the center of the pattern image (in the middle of the gunshot hole, if there is one), and the number of concentric rings, inside which the optical density is calculated using Equation (11). The pattern image is close to centrally symmetric with respect to the gunshot hole. Therefore, we consider circles, rings with their center in the middle of the gunshot hole, and sectors as areas of interest. Choosing the areas in the form of concentric rings and sectors, one can experimentally determine the GSR distribution in the cross section of the gas–gunpowder jet.
(iii) The integrated optical density in the areas of interest is determined by the formula
  (11) $$D_{\Sigma }=-\ln\frac{\Sigma_{i}I_{i}}{I_{0}N},$$
  where i is the pixel number and $I_{i}$ is the brightness value in the pixel.
(iv) The GSR mass on the selected area is estimated by Equation (10).
(v) Diagrams of the GSR distribution over the target surface are plotted.

In these calculations, the coefficient ${\beta /\sigma }_{i}$ is still undetermined. The pixel area $\sigma_{i}$ is determined as the product of optical magnification used in taking the photo-image and the nominal value of the pixel size of the photomatrix. In practice, the pixel size is calculated from a scale bar photo-image (ruler image). The value of β can be determined in experiments. In practice, the mass in grams can be obtained by means of preliminary absolute calibration of the method on the basis of results of an independent method of finding the GSR mass $m_{cal}$ on standard targets, as described further in Section 5.

**Figure 1:**
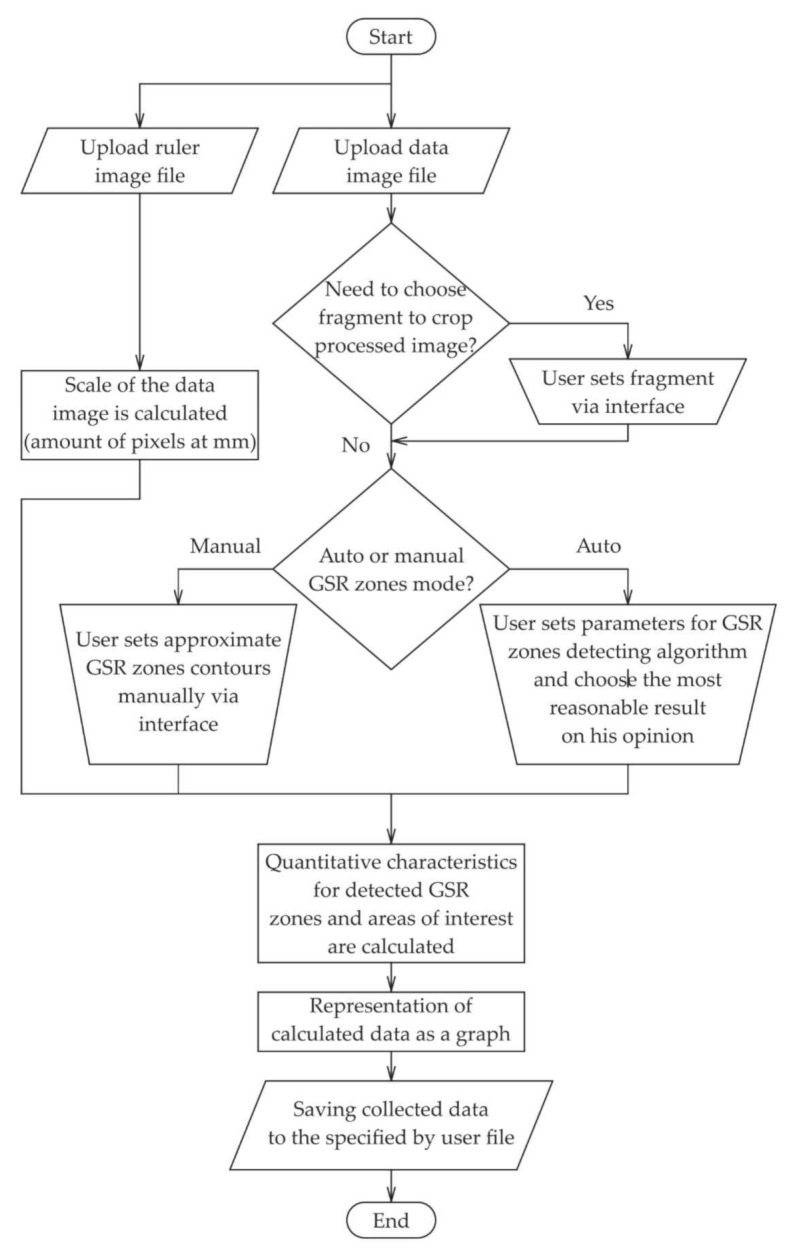
Workflow of ImgOpinion application.

We developed specialized software named ImgOpinion, which performs optical–structural analysis of digital images. The expected workflow when using the developed application is shown in the flow chart in Figure 1. The management of the calculations in the module “quantitative characteristics for detected GSR zones and areas of interest are calculated” is explained via the pseudocode Algorithm 1 for calculating the average optical density pixel by pixel within an area of interest defined by two contours.

Pseudo code algorithm for calculating the average optical density pixel by pixel within an area of interest defined by two contours:


**Algorithm 1.** Pseudo code algorithm for calculating the average optical density.
**algorithm** average-density-calculation **is** **input**: source image *srcImg* with colors at HSV color space, outer contour of ROI *outerContour*, inner contour of ROI (must be contained inside *outerContour*) *innerContour* (by default *innerContour* is null)    average brightness value (V from HSV) of previously selected via application interface image area not covered by soot *cleanValue* **output**: average density value *area* ← 0    *sumDensity* ← 0    **for each** pixel *p*
**in**
*srcImg*
**do**      **if**
*p* inside *outerContour*
**and**
*innerContour is null*
**or**
*p* inside *outerContour*
**and** outside *innerContour*        *area* ← *area* + 1        *currentValue ← brightness value (V from HSV) of p*        *currentDensity* ← ln(*currentValue/cleanValue*)        *sumDensity* ← *sumDensity* + *currentDensity*    **return**
*sumDensity*/*area*


ImgOpinion is a desktop application designed for use at expert laboratories. The application is written in Java programming language and runs on systems of Linux and Windows families. The system requirements of the ImgOpinion application on Windows are the same as those of Adobe Photoshop (Adobe System Incorporated [36]). The choice of the programming language was made because Java has convenient tools for desktop applications development, and it is a cross-platform programming language itself. For image processing, OpenCV [37] library was chosen because, firstly, it has better performance compared to other libraries, which provide similar functionality [38], secondly, a lot of various tools are available for using OpenCV with other technologies, including Java programming language, and finally, OpenCV is an open-source programming product and is free for use under the open-source Apache 2 License [39]. The application can proceed with images that are not larger than 2^30 pixels. Currently, the following image file formats are supported: Windows bitmaps—*.bmp, *.dib, JPEG files—*.jpeg, *.jpg, *.jpe, Portable Network Graphics—*.png, TIFF files—*.tiff, *.tif.

**Figure 2:**
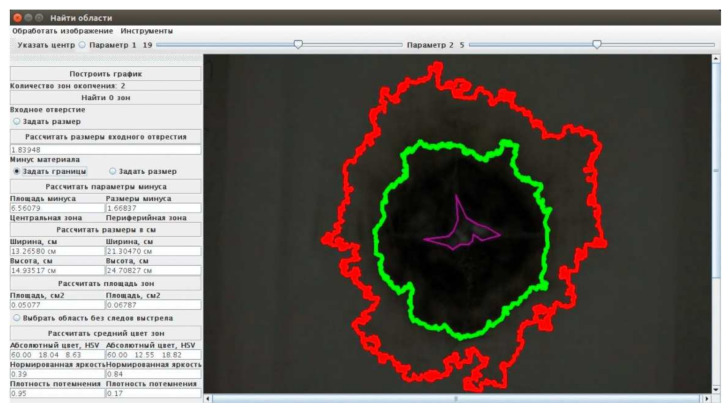
Dialog box of ImgOpinion application.

The window of ImgOpinion application in “the automatic GSR zones search” mode is shown in Figure 2. The program calculates the parameters of the morphology of the shot marks and the topography of their deposition and shows the contours of the GSR deposition zones, determined using the Otsu segmentation algorithm [40].

## 4. Experimental Results

The general course of the experiment includes the following steps: step 1—making targets and shooting on a special stand; step 2—images acquisition of targets with GSR; step 3—image processing, including preprocessing and ImgOpinion operation; step 4—analysis of the output data.

Step 1. Experimental objects were prepared for the forensic ballistic examination of gunshot marks, namely targets measuring 300 × 300 mm made of light-colored fabric of various densities. Shooting was carried out from close distances of 3, 5, 7, 10, 15, 20, 30, and 40 cm from the 5.45 mm AK-74M assault rifle with 5.45 × 39 mm (7N6) cartridges, 9 mm Makarov pistol with 9 × 18 mm PPO cartridges, and 7.62 mm TT pistol with cartridges of 7.62 × 25 mm. At each range, three targets with GSR were obtained for each type of small firearm. Below are some experimental results for targets (samples) made of white coarse calico. Sample 1 contains GSR at a distance of 5 cm (TT pistol). Samples 2, 3, and 4 contain GSR at distances of 5, 15, and 40 cm, respectively (AK-74M).

Step 2. When processing the target images, calculations are performed with the brightness values in pixels. Therefore, in the experiment, it is necessary to pay attention to the choice of an illuminator. We used two different illuminators. Images acquisition “in reflected light” was carried out with the multifunctional semiconductor illuminator “Photobox 3138” [41,42]. The lighting variation does not exceed 2% at the edges of the working field of 300 × 300 mm. The “Photobox 3138” design includes a white LED illuminator with a color temperature of 5000K (CIE D50) and a high color rendering index (CRI 97+). Images acquisition “in transmitted light” was carried out with LMPRS Office Slim 15 315 LED lamp (produced by Lampiris plant, Novosibirsk, Russia) by a Canon EOS 500D camera (produced by Canon Inc., Tokio, Japan).

In the experiment, the attention has to be paid to the choice of camera. The specific features of camera selection and its setup modes for the acquisition of images, which are used to calculate the optical density in pixels of the resulting digital image, are discussed in detail in [27]. In [27], a method for camera calibration is also presented and test objects are proposed, experimental results of various cameras testing are given, and a comparative analysis of them is carried out.

The Canon EOS 500D camera allows a user-tuned regime where image preprocessing by the built-in processor of the camera itself is canceled, while the white balance set by the user makes it possible to avoid automatic correction of the ratio of color channels. This regime implies manual setting of the white balance, absence of balance shifting, absence of bracketing, “exact” image style, light sensitivity ISO 100, no extension of the ISO range, no noise suppression in the case of long exposure and high ISO values, no priority of colors, and no automatic correction of brightness.

The image was taken in the regime of user-defined tuning of the camera in the RAW 14-bit format, which was then converted to the TIFF 16-bit format. The 16-bit TIFF format does not limit the number of brightness gradations contained in a 14-bit image. In our experience, in addition to the TIFF format, PNG and DICOM formats are also suitable for working with 16-bit digital images. These formats do not introduce additional distortions into the original image. For professional cameras, there is usually software that converts the internal RAW format into TIFF or PNG formats. In addition to preserving high image quality, TIFF is an adaptable format that can support both lossy and lossless compression.

Step 3. The recorded images of the samples were pixel-by-pixel normalized to the corresponding images of the white background sheet captured by the cameras beforehand. Such normalization prevented vignetting and possible nonuniformity of field illumination.

These normalized images were used for all calculations in the ImgOpinion application. To eliminate the inherent noise (a target fabric texture), the digital images of samples were preprocessed by a smoothing spatial filter [20]. Noise reduction can be accomplished by blurring with a linear filter and also by nonlinear filtering. We used an image smoothing with Gaussian mask of 3-pixel size.

**Figure 3:**
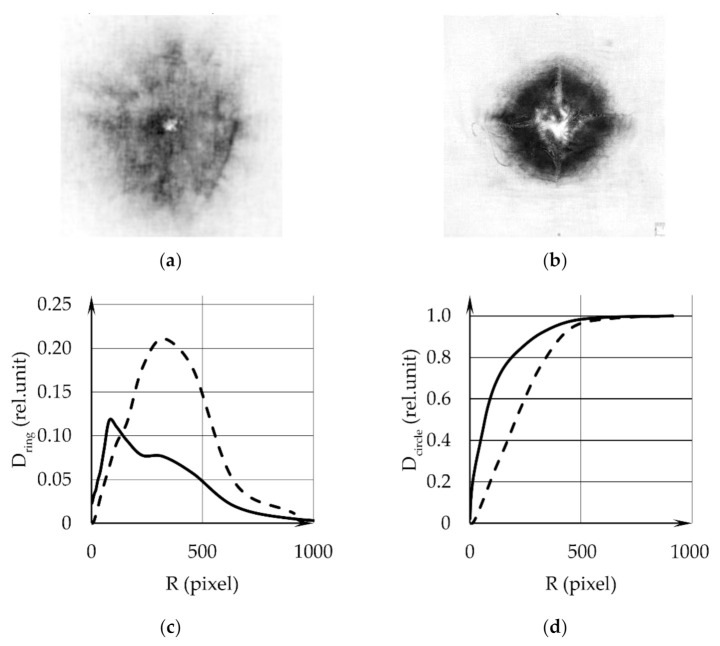
Experimental results for sample 1 and sample 2: (**a**)—the monochromatic photo-image of the sample in transmitted light (the image of sample 1); (**b**)—the image of sample 2; (**c**)—the integrated optical density D_ring_ in the rings versus their outer radius R, the solid line refers to sample 1, the dotted line—to sample 2; (**d**)—integrated optical density D_circle_ in a circles versus their radius R, the solid line refers to sample 1, the dotted line—to sample 2. One pixel contains 84 microns.

Step 4. The images of samples 1 and 2 are shown in Figure 3a,b. During the mathematical processing, annular regions were selected in each digital image. The diagrams for the dependence of the integral optical density D_ring_ in the rings as a function of their outer radii (Figure 3c) display the GSR distribution (GSR layer thickness) over the surface of the samples versus the distance from the center. The dependences of the integral optical density in a circle D_circle_ on its radius R are given in Figure 3d. The solid lines in Figure 3c,d are the data for sample 1, and dotted—for sample 2. The diagrams reveal and give the possibility to quantitatively characterize the differences in GSR distributions calculated with the digital images of different samples obtained with weapons of different types (TT or AK) at the same distance.

**Figure 4:**
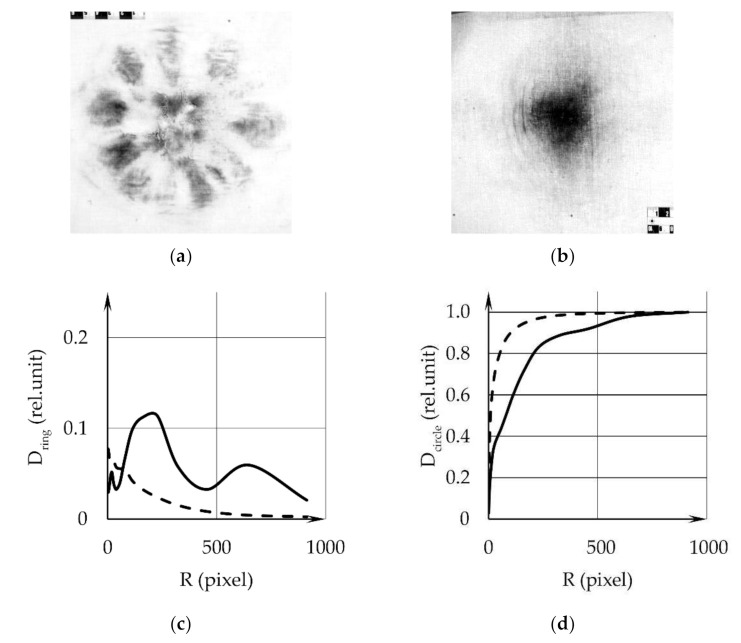
Experimental results for sample 3 and sample 4: (**a**)—the monochromatic photo-image of the sample in transmitted light (the image of sample 3); (**b**)—the image of sample 4; (**c**)—the integrated optical density D_ring_ in the rings versus their outer radius R, the solid line refers to sample 3, the dotted line—to sample 4; (**d**)—integrated optical density D_circle_ in a circles versus their radius R, the solid line refers to sample 3, the dotted line—to sample 4. One pixel contains 84 microns.

Figure 4a,b shows images of samples 3 and 4. The diagrams for the dependence of the integral optical density D_ring_ in the rings on their outer radii (Figure 4c) display the GSR distribution (GSR layer thickness) over the surface of samples, versus the distance from the center. The dependences of the integral optical density in a circle D_circle_ on its radius R are given in Figure 4d. The solid lines in Figure 3c,d are the data for sample 3, dotted—for sample 4. The graphs reveal and give the possibility to quantitatively characterize the differences in sGSR distributions calculated from the digital images of different samples obtained with weapons of the same type (AK) at different distances.

The analysis of the optical density in concentric rings in the images reveals the radial dependence of the GSR distribution in the cross section of the gas–gunpowder jet.

**Figure 5:**
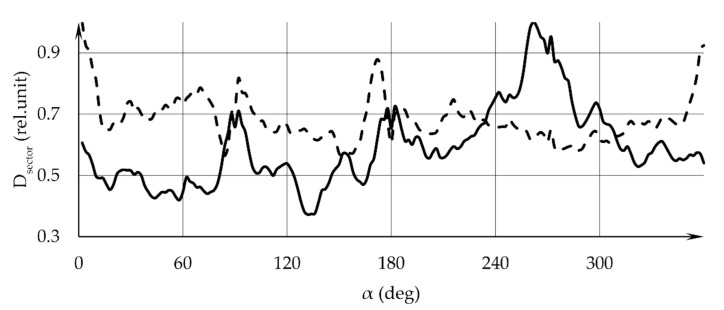
Angular distribution of the integrated optical density D_sector_ over the samples’ surface. The solid line refers to sample 1, the dotted line—to sample 2. α—angular coordinate in the sample plane.

**Figure 6:**
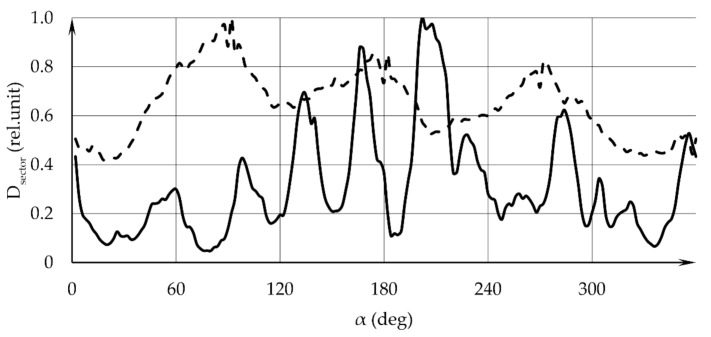
Angular distribution of the integrated optical density D_sector_ over the samples’ surface. The solid line refers to sample 3, the dotted line—to sample 4. α—angular coordinate in the sample plane.

Additional information is provided by the analysis of the angular distribution of the GSR layer thickness on the target, which is performed by means of calculating the integrated optical density in sectors of a selected circle in the sample image. The circle center coincides with the center of the pattern image, and the circle is divided into sectors with a specified angular size. The circle radius is prescribed and can cover the entire visible area covered by GSR. The results of calculating the integrated optical density in sectors with an angular size of 2 degrees are shown in Figure 5: the solid line refers to sample 1, the dotted line—to sample 2. In Figure 6, the solid line refers to sample 3, the dotted line—to sample 4. The angular dependence of the optical density manifests itself in the form of oscillations in the diagrams in Figure 5 and Figure 6. Peaks are achieved at the same angular coordinate values for all sector opening angles used.

**Figure 7:**
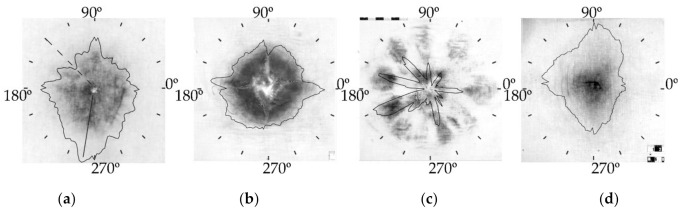
Angular distribution of the integrated optical density D_sector_ over the samples’ surface in the form of the indicatrix: (**a**)—sample 1; (**b**)—sample 2; (**c**)—sample 3; (**d**)—sample 4. The direction of the maximum optical density is shown by the solid line; the direction of the minimum optical density is indicated by the dotted line.

It is convenient to represent the angular distribution of the soot layer thickness on the sample in the form of an indicatrix, as shown in Figure 7a–d.

In Figure 7, indicatrices are superimposed onto images. In Figure 7a, the solid radial line indicates the direction of the sector with the maximum optical density, and the dotted line—the direction of the sector with the minimum optical density.

The analysis of the optical density of the image in the selected sectors of the circle reveals the angular dependence of the GSR distribution in the gas–gunpowder jet cross section. The indicatrices visually represent and allow quantitative characterization of the differences in the angular distributions of the GSR layer thickness calculated from the images of various samples obtained both with weapons of different types (TT or AK) at the same distance and with weapons of the same type (AK) at different distances.

## 5. Discussion and Conclusions

The proposed approach and the present consideration allow one to detect and experimentally determine the GSR distribution in the cross section of the gas–gunpowder jet. By choosing the areas of interest in the digital image of the pattern in the form of concentric rings, circles with increasing radii, and circular sectors, and calculating the optical densities in these areas, one can find both the differential and integral distributions of substance in the cross section of the gas–gunpowder jet, which is related to the firearm type and to the details of using this weapon. Results obtained in the study confirm the possibility of deriving integral and differential regularities of a GSR distribution over the surface of targets made of different materials. The shot distance, gunpowder type, and weapon type directly affect these distributions. The research we carried out proves the assumptions made in [43]. Thus, using the distribution function of GSR on the target surface, one can determine the shot distance.

The proposed approach is justified with the use of some approximations and constraints. All images have to be taken under the same lighting conditions, using the same camera with identical tuning which does not distort the color reproduction, brightness correction cannot be performed, and quantitative photogrammetry must be allowed for.

The approach can be applied to targets in the case of close-distance shooting [44] in the region of priority deposition of GSR. It was assumed in the present consideration that the gas–gunpowder jet completely transferred soot to the target surface. In this case, the mass of matter deposited on the target is expected to be independent of the distance to the weapon muzzle. At large values of the GSR layer thickness, the light absorption coefficient is independent of the GSR layer thickness, and the present approach does not yield the expected results. The marginal thickness of the GSR layer can only be determined experimentally.

It is also relevant that coefficient *β* is not completely defined in the analysis. Little information is available about coefficient *β*. Apparently, this coefficient depends on the gunpowder type, on the gunpowder absorption coefficient, and on the concentrations of metal and oil inclusions. Additional experimental data are needed here. It is possible to overcome this uncertainty by calibrating the measurement method.

As applied to the case considered in the present paper, this means that there should exist another (independent) method of finding the soot mass $m_{cal}$ on the target, which should be used for calibration in the following way. Shooting is performed on standard targets at standard distances $\left(l_{1},l_{2},l_{3},l_{4}\ldots \right)$ from the weapon muzzle. Digital images of the patterns are taken. The GSR mass on each target $\left(m_{cal1},m_{cal2},m_{cal3},m_{cal4}\ldots \right)$ is found by using the independent method. The optical densities $\left(D_{\Sigma 1},D_{\Sigma 2},D_{\Sigma 3},D_{\Sigma 4}\ldots \right)$ and masses $\left(m_{cal1},m_{cal2},m_{cal3},m_{cal4}\ldots \right)$ are calculated on the basis of these images. The calibration equation $m_{cal}\left(m\right)$ is determined for different distances l. If there is no GSR deposits, then m=0 and $m_{cal}=0$. Thus, the dependence $m_{cal}\left(m\right)$ is a straight line passing through the origin and a nonzero point (which was determined). If it is possible to determine nonzero points for different distances l, then the slopes of the lines provide an idea about the shot distance. Apparently, the calibration lines will be slightly different for different weapon and gunpowder types. Thus, an experimental determination of the coefficient $\beta /\sigma_{i}$ is actually carried out.

After that, one can study the working target and apply the calibration dependence (found values of $\beta /\sigma_{i}$) to determine the GSR mass in the international system of units (SI).

In the present study, we developed a method of mathematically processing the digital images of patterns and provided the theoretical and mathematical justification and the experimental validation of the possibility of estimating the amount of GSR and determining GSR distribution over the target on the basis of its digital image. The analysis of the optical density in concentric rings in images reveals the radial dependence of soot distribution in the cross section of a gas–gunpowder jet. The analysis of the optical density in the selected sectors of the circle reveals the angular dependence of the soot distribution in the gas–gunpowder jet cross section.

It is possible to quantify the differences in the radial and angular distributions of the thickness of the GSR layer on various targets obtained both with the help of weapons of different types at the same distances and with the help of weapons of the same type at different distances by calculating the distribution of optical density on their digital images.

The developed method creates the necessary prerequisites for optimization of the forensic ballistic expert examination regarding organization of the expert experiment and reduction of time and material expenses for its production, providing the expert with the information received by calculation about a possible interval of the required distance of the shot.

The prospect for the development of the obtained scientific results is determined by the creation, in the future, of information reference databases of traces of close shots fired from various models of firearms, their analytical processing by the mathematical apparatus, and the establishment of appropriate correlations between the calculated characteristics of gunshot traces and distances of the shot at which they were formed.

## Data Availability

Data are available upon request to the authors.

## Glossary

**Symbol Name**	**Meaning**
*R*	The intensity reflection coefficient
*A*	The intensity absorption coefficient
*T*	The intensity transmission coefficient
*β*	A proportionality coefficient, which characterizes the properties of the target material, gunpowder, and its adhesion to the target and which is expected to depend on the light wavelength
*l*, *l*_1_, *l*_2_…	Standard distances from the weapon muzzle, number 1, 2, 3… means the target number
*m*, *m*_1_, *m*_2_…	The mass of the GSR on the selected area, number 1, 2, 3… means the target number
*m_cal_*, *m*_*cal*1_ …	The soot mass on standard targets, number 1, 2, 3… means the target number
*N*	The number of pixels in the selection area
*R*	The reflection coefficient in terms of intensity
$\tilde{S}$	The selected area
*i*	The pixel’s number (e.g., a subscript)
*T_i_*	The intensity transmission coefficient in pixel
*D_i_*	The optical density in pixel
*D*_Σ_	The integrated optical density in the areas of interest
*D*_Σ1_, *D*_Σ2_,…	The integrated optical density in the image, number 1, 2, 3… means the target number
*h*	The GSR layer thickness
*h_i_*	The GSR layer thickness in pixel
*h_max_*	The maximum GSR layer thickness in pixel
*h_eff_*	The effective thickness of GSR
*I_i_*	The local brightness in pixel
*I*_0_	The brightness (mean value) in the image in the regions where GSR is absent
σ	The surface area
*σ_i_*	The area of one pixel
*dI_i_*	The brightness differential in pixel
*dh_i_*	The layer thickness differential in pixel
